# Supplementary figures and images for: DOC export is exceeded by C fixation in May Creek: A late-successional watershed of the Copper River Basin, Alaska
Source: PLoS One. 2019 Nov 20;14(11):e0225271. doi: 10.1371/journal.pone.0225271 (PMC6867643; doi:10.1371/journal.pone.0225271)

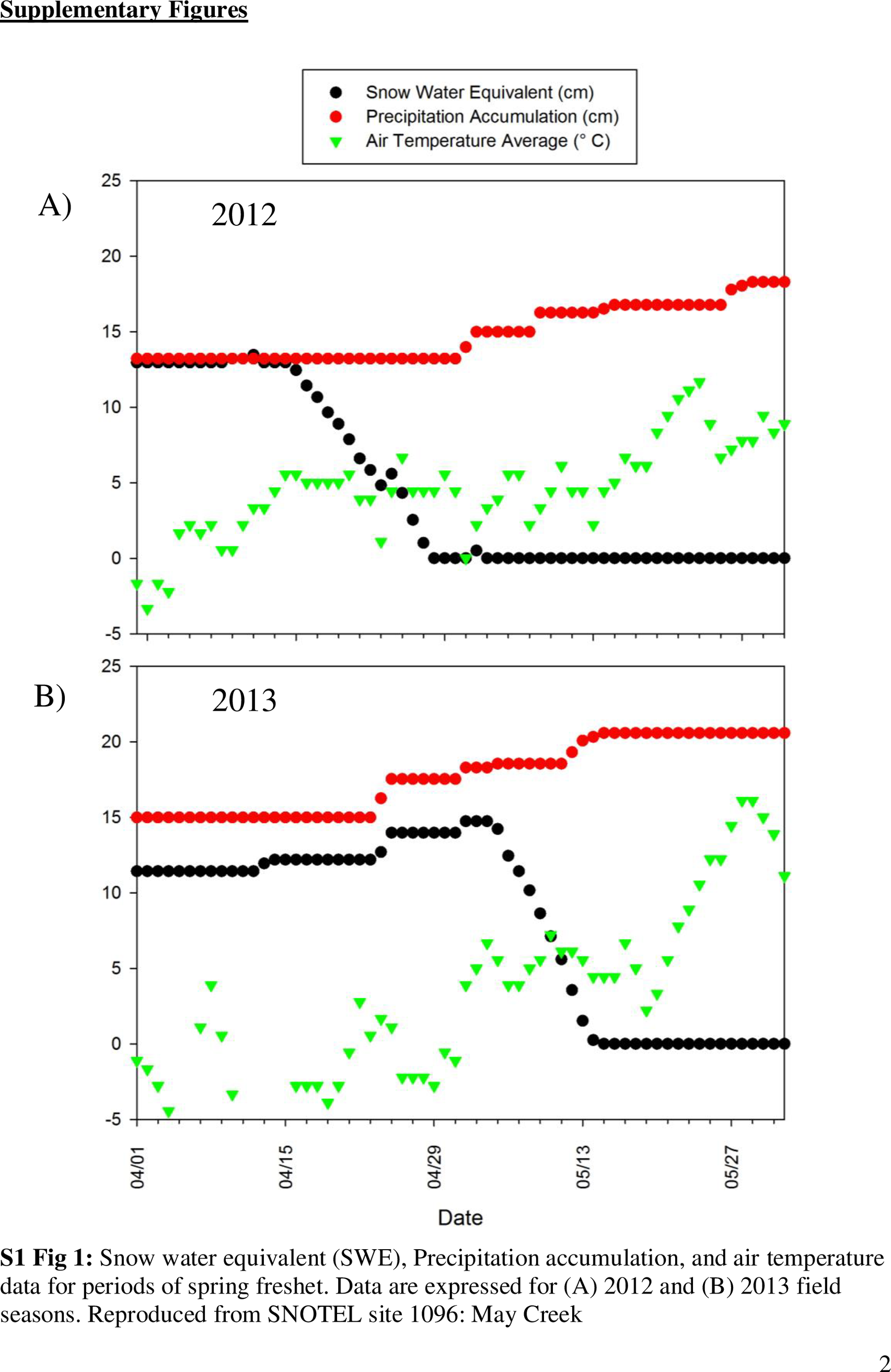

Supplement: S1 Fig — Data are expressed for (A) 2012 and (B) 2013 field seasons. Reproduced from SNOTEL site 1096: May Creek. (TIF) [file pone.0225271.s001.tif]

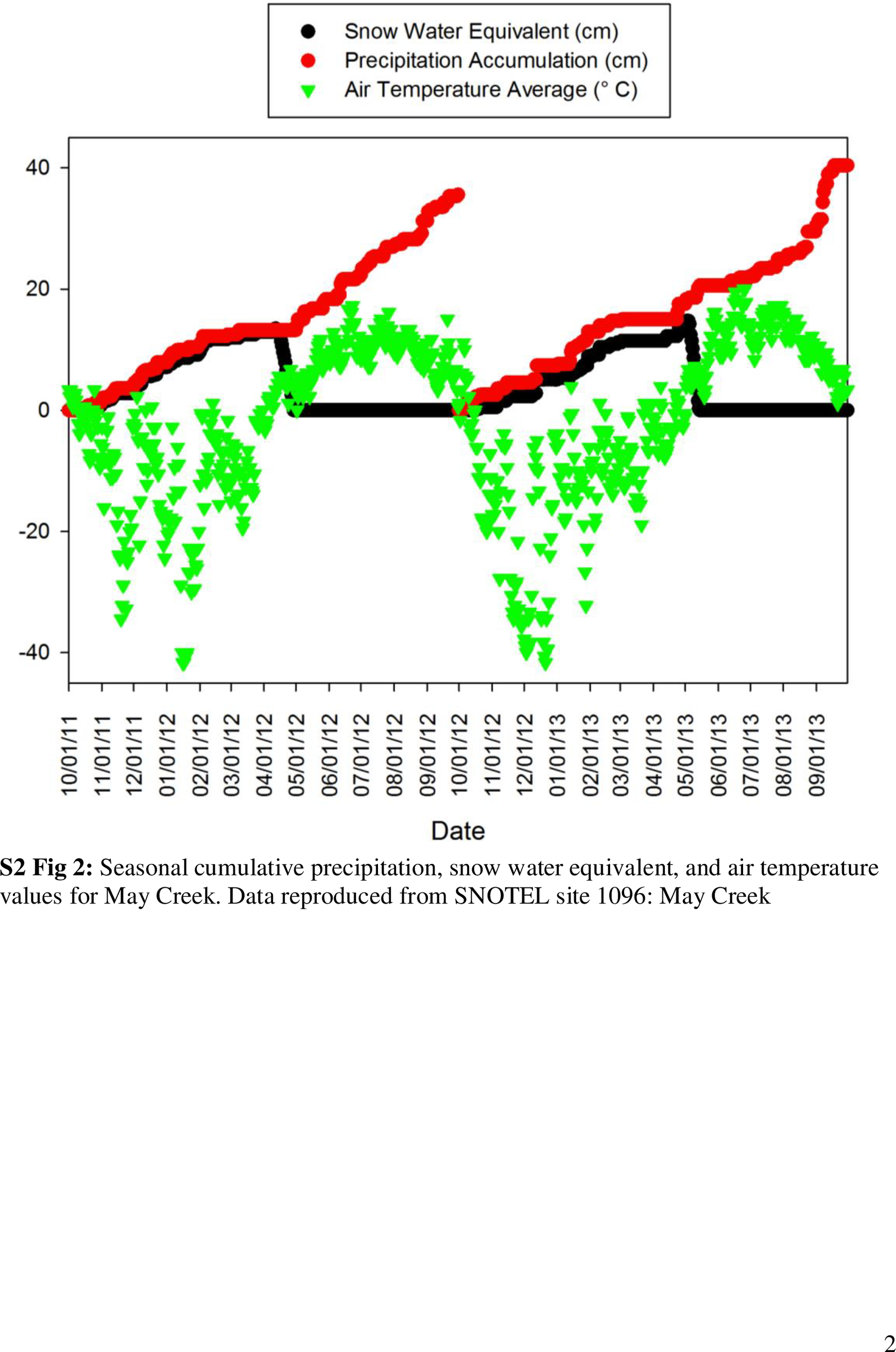

Supplement: S2 Fig — Data reproduced from SNOTEL site 1096: May Creek. (TIF) [file pone.0225271.s002.tif]

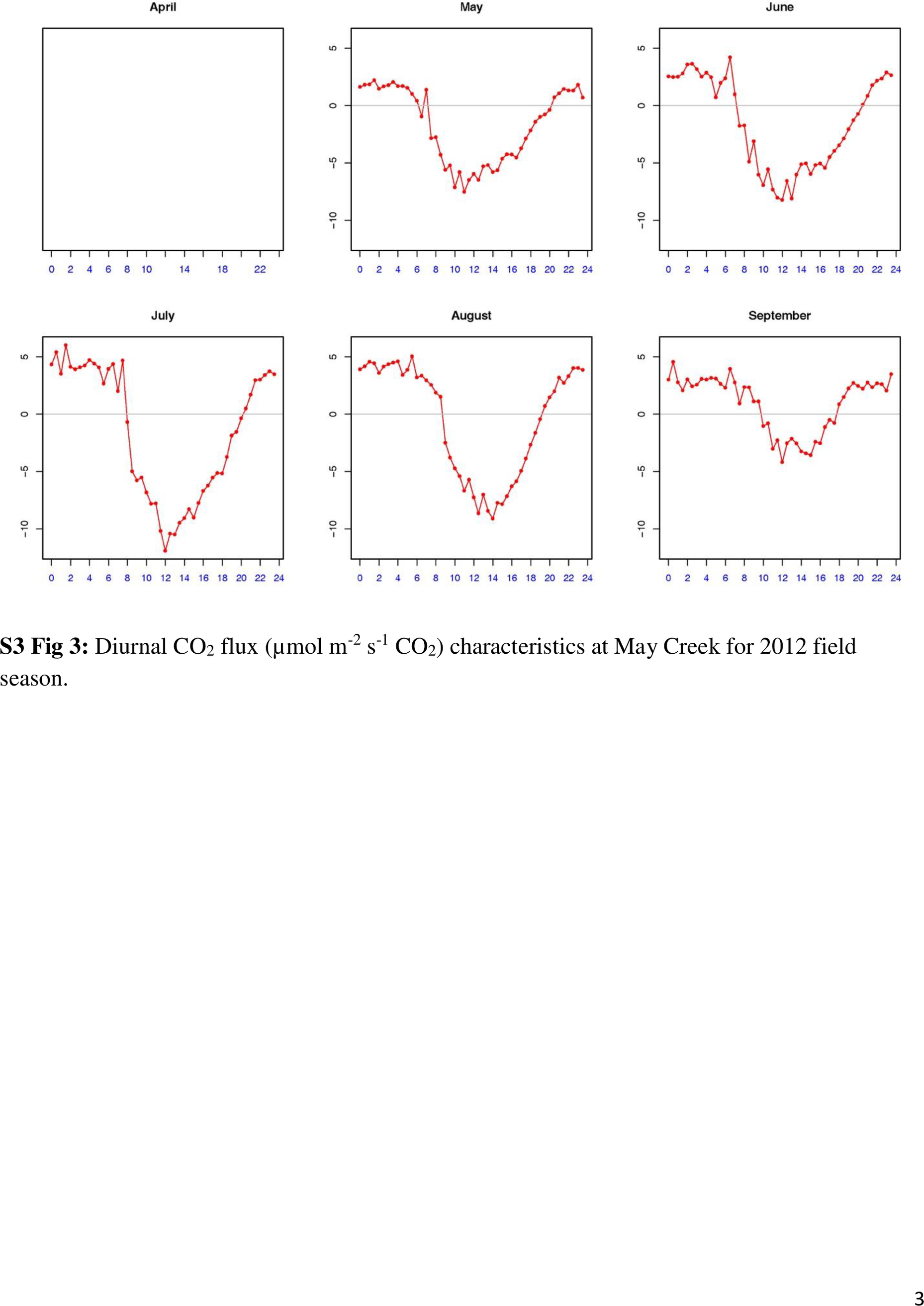

Supplement: S3 Fig — (TIF) [file pone.0225271.s003.tif]
