# Supplementary material for: DOC export is exceeded by C fixation in May Creek: A late-successional watershed of the Copper River Basin, Alaska
Source: PLoS One. 2019 Nov 20;14(11):e0225271. doi: 10.1371/journal.pone.0225271 (PMC6867643; doi:10.1371/journal.pone.0225271)
Supplement: Attachment — Submitted filename: Response to Reviewers.docx [file pone.0225271.s009.docx]

**Comments from the Editorial office:**

1. You may seek permission from the original copyright holder of Figure [1] to publish the content specifically under the CC BY 4.0 license.
2. If you are unable to obtain permission from the original copyright
   holder to publish these figures under the CC BY 4.0 license or if
   the copyright holder’s requirements are incompatible with the CC BY
   4.0 license, please either i) remove the figure or ii) supply a replacement figure that complies with the CC BY 4.0 license.

*(Combined response to these comments) We have removed Figure 1 and replaced it with a site map of the same area using the USGS NED 1/3 arc second DEM at ~10 meters resolution and the USGS National Hydrography Dataset for the flowpaths. Both of these are freely available online and there should be no copyright concerns.*

3. Please include captions for your Supporting Information files at the end of your manuscript, and update any in-text citations to match accordingly.

*We have included captions at the end of the manuscript as requested.*

4. Please include in the Methods section the names of the locations, and provide geographic coordinates for the data set.

*We have provided the location names and GPS coordinates of each in the Methods section.*

**Review Comments to the Author**

Please use the space provided to explain your answers to the questions above. You may also include additional comments for the author, including concerns about dual publication, research ethics, or publication ethics. (Please upload your review as an attachment if it exceeds 20,000 characters)

**Reviewer #1: GENERAL COMMENTS:**

The manuscript provides a useful carbon dataset that helps link forest/overland carbon flux with stream export of dissolved organic carbon (DOC). These data and findings will be of interest to a broad audience working on global to boreal carbon cycles. The authors present an interesting story around seasonal drivers of carbon flux and help highlight how growing condition (light and temperature) and water (precipitation and snowmelt) influence differently dissolved and gaseous carbon flux. The manuscript has many positives and I think a few modifications are needed to help the manuscript more fully explore the data and bring together the full story. I have three main suggestions that I think would make the manuscript more useful.

1) Make full use of the EEMs --- Given that you have the full EEM, why only focus on FI and HIX. Peak picking (Paulo Coble's work) or PARAFAC might provide more useful insight when tested through a multivariate DOM method. SUVA and spectral slope would also be useful measure to understand how the complexity of DOM changes across the growing season. The discussion starts to exam the EEMs visually, but I think it would be useful to take the analysis farther by using the full set of DOM parameters that optical chemistry can generate. If these data are then analyzed through multivariate analysis, one can speak about the DOM as a pool and detailed univariate explanations of all the variables could be avoided.

*We thank the reviewer for these comments and have made extensive revisions to our use of the EEMs dataset. We have re-analyzed our data and have been able to fully-validate a 3 component PARAFAC model. We have identified these components in context of peak-picking (C,M, and T as the reviewer mentions) and identified a strong correlation between Component 1 and HIX along with a very strong inverse correlation between Component 3 and HIX. The manuscript is updated to identify how these components vary seasonally and between sites. We have also drawn comparisons of our study’s DOM characteristics to those found in other studies through the OpenFluor database. We will upload our study’s EEMs to this database to allow it to be searchable by others.*

2) Statistics, questions, and hypothesis tested needs more detail and justification --- It is unclear to me what test and question were used to generate p-values in this study. More detail is needed for the reader to understand what data are being compared, why, and how. For example, how did you define season? How are you testing rate of change across time? I think better defining the studies hypothesis and question with the link statistics will make the results easier to follow with respect to your broader story.

*We thank the reviewer for requesting we clarify our statistical methods. Our temporal trends were assessed using linear regression and analysis of covariance (ANCOVA) for slope homogeneity. This test is recommended by EPA for temporal trend watershed comparisons,* [*https://www.epa.gov/nps/nonpoint-source-monitoring-technotes*](https://www.epa.gov/nps/nonpoint-source-monitoring-technotes)*. We have re-assessed our word choice in the manuscript and provided clarifications throughout to indicate elsewhere which specific analyses are performed.*

*Our seasons were defined by the bracket of time our field crew was available to deploy sensors and collect grab samples. In 2012 and 2013 this was May-Sept, with the exact dates slightly deviating based on field logistics. As with the previous comment, we have extensively re-worded the manuscript to provide clarifications on these aspects and linkage of the statistical comparisons to hypotheses.*

3) Combining results and discussion sections will make it easier for your reader to follow the data and the story --- Overall the results are written clearly and as individual statements I understood the pattern and result being described. I think what I struggled to understand was how all the variables and different temporal scales that where tested fit together as one story. The discussion and abstract did a fantastic job describing the overall story but at times I struggled to link the broad story to the specific results. I think to help the reader move through your complex data set, combining the results and discussion section would communicate more clearly the big picture story and the result that supports that big idea. This might shorten then manuscript a little as well better highlight the interpretation of the result alongside the result.

*Thank you for noting this, we have extensively re-formatted the results section and discussion section into a combined “Results and Discussion” that integrates the components mentioned. The appropriate discussions have been paired with the findings and we believe that the manuscript reads more fluidly now. We also appreciate the reviewer acknowledging the complexity and nuances of this dataset; we sincerely appreciate these constructive suggestions! It really helps us make the paper better!*

SPECIFIC COMMENTS

Abstract: The abstract is clear and provides a nice snapshot of the study and its relevance. I did not fully understand the last sentence of the abstract. I am not certain what shift is being referenced and I am not certain in what respect this carbon mass is integrated and important. Could the sentences be revised to be more exact?

*Thank you for the compliment on the abstract! We updated the wording to “climate change-induced seasonal shift” to clarify that is what we are referring to.*

Key Points:
1) Does this include the frozen period? It seems odd that CO2 fixation wouldn't change between growing and dormant seasons. This point might need to be qualified with the timer period of the study

*Thank you for catching this, we updated the wording to “annual C sinks” to indicate that this timeframe is integrated over a whole year.*

3) Browning has multiple means. Since your paper is about DOC, could you use another word to describe the drought impact on the forest?

*We removed the specific term “browning” and left the general term “drought-induced”, which is a broader yet more accurate description.*

4) Did you mean carbon or DOC rather than nutrient?

*Yes; we updated this as suggested.*

Introduction: the introduction is framed well and does a nice job setting up the study and the need for the combined study of carbon gas and DOC flux. The introduction was a little long. You could likely remove the paragraph about DOM fluorescence without loss to your overall story. To me this approach is now very common and it's OK to not focus on how DOM was characterized but focus instead on what knowledge around the DOM characteristics can tell us about C cycles in this study

*Thank you for this suggestion. We have eliminated several sentences from this paragraph and focus more on contextualizing previous approaches employed in similar systems.*

L150-L155: I think MC needs to be define in text. MC could also be used when describing the actually creek in text as it was in the figures

*We updated this to define May Creek as MC and checked the manuscript to ensure the term is being used as the reviewer suggested.*

L189: Did you mean fluorescence rather than absorbance?

*Yes thank you for catching this; we corrected absorbance with fluorescence*

L338-L339: The July value is nearly 3x higher. This seems a much larger change in magnitude than the water isotopes and fDOM, FI, and HIX values above, which were interpreted as significantly different. Why is this considered stable?

*This was an oversight; we removed the descriptive term “very little” from this section and simply note the range of values.*

L444: This should be a statistical inspection using multivariate methods to show how the DOM pool changes. These visuals help show the pattern, but I think the data need to be analyzed statistically, in order to show the magnitude and direction of change.

*We have added significant new statistical analyses to this section, a validated 3 component PARAFAC Model. The three components represent typical “A, C, M, and T”-like fluorescence widely described across the literature. We demonstrate component 1 is highly correlated with HIX and component 3 is inversely correlated, which is consistent with our previous discussion. We updated the manuscript to indicate the seasonal fluorescence trends are relatable to HIX. Also, we uploaded our data to OpenFluor, and matched our fluorescence components to those in the database, noted elsewhere in the literature. This context has been added to the manuscript and we are confident this has greatly improved the interpretive power and applicability of our dataset.*

L459-L462: This hypothesis needs to be stated in the methods with statistical test explaining how the hypothesis was tested.

*We updated the methods with this information as requested by the reviewer.*

Figure 5: Could error bars be added to each point to show how much day to day or night to night variation is displayed in each monthly mean at each time point?

*Thank you for this comment, these were point values without error bars.*

Figure 7: These are useful plots. I think the data within the EEMs needs to be explored in the results section through PARAFAC or peak picking. Otherwise, this rich data set isn't explored to its fullest. In terms of the plot, the xyz axis font is to small and not a very good resolution. I zoomed in and the text was fuzzy and difficult to read. You should make a note that the z-scales vary from plot to plot and specify the units (RU, QSU, etc...). Conversely, you could standardize each plot by the max value and this would allow all plots to be displayed on the same relative scale. The second option would draw the reader to changes in characteristics of DOM between spring and fall rather than highlight changes "concentration"

*Please see previous comment. Figure 7 has been deleted and replaced with a figure depicting the 3-component PARAFAC model, which provides more robust statistical handling of the fluorescence data in comparison with the visuals. We made sure that the figure is in sufficient resolution for viewers to interpret. Thank you again for these suggestions, it has greatly improved the quality of this manuscript!*

**Reviewer #2: Review PlosOne.**

This manuscript presents the results of a two-year study conducted in a boreal watershed of Alaska. The authors describe the fluxes (magnitude, seasonal and annual variations) and chemical characteristics of DOC in four stream types of watersheds characterized by different vegetation cover and soil characteristics. They also measured CO2 fluxes for one growing season at one of the sites to assess the significance of DOC exportations as compared to C fixation through photosynthesis. This study was motivated by the fact that global warming may increase DOC outputs and reduce CO2 fixation in these ecosystems, which may turn them into C sources. This is an interesting and important topic that researchers must tackle. The main conclusion is that the watershed does not export large amounts of DOC as compared to C fixation. Therefore, in contrast with their hypothesis the watershed is still a C sink and highly C conservative. They also found that most DOC efflux occurred as a pulse during snow melt in May.
The amount of work, the sampling effort (four streams), the methods (eddy covariance, fluorescence spectroscopy) and analyses (stable isotopes, DOC concentrations) deployed for this study are considerable. The authors described the materials and methods thoroughly and clearly, and the bibliography is thorough.

*We thank the reviewer for identifying the key points and hypotheses of our study and for the compliment on the breadth of our dataset, description of methods, and bibliography! This reviewer provided extensive comments that has greatly improved the quality of our manuscript.*

The results are interesting and deserve publication. However, I was disappointed by the way the authors presented, interpreted discussed their results. First, the figures could be of better quality. I think for instance that the use of dots instead of line prevents from visualizing the seasonal trends. The resolution of Fig 7 is too low for the x- and y-labs to be readable. I think that the figures should also be reorganized to facilitate the discussion. For instance, why not grouping results with similar trends in a single figure (e.g., fDOM with DOC flux, and delta 18O with Q)?.

*Thank you for identifying and suggesting several ways that we can improve the visual presentation of our data. As mentioned with our responses to reviewer #1, Figure 7 has been deleted and replaced with a new figure describing the PARAFAC model; it is of sufficient print quality and can be seen more easily. In regards to the organization and grouping of figures, as the reviewer notes, the dataset is complex and nuanced. The selection of appropriate data to present in each figure is difficult and we carefully assessed which visual pairings would complement the text body in the clearest manner. We decided to group figures according to the information presented in its corresponding paragraph. Many of these components are complimentary as the reviewer points out, and additional cross-correlations are possible. However, due to the breadth of the material we felt it best to prioritize groupings this way. We have provided the data with the manuscript and viewers who wish to visualize it in different ways are free to explore it as they see fit.*

The authors insist on the importance of precipitation and temperature on DOC fluxes in this type of ecosystem but did not include the data with the ms and didn't discuss their results properly. I would bring some of these data from the supplementary materials to the main text and maybe remove the Fig 5 which does not provide particularly insightful information. The fact that the vegetation is a source of CO2 at night and a sink during the day is quite obvious.

*We thank the reviewer for their concerns; we can see how Figure 5 may not be worth prioritizing in the figure list. Since we refer to it in the text, we moved Figure 5 to Supplemental Information. In regards to whether precipitation and temperature data were included with the manuscript, we respectfully note that these were provided in the supplemental information section and are freely available for viewing; data were provided from a nearby SNOTEL site and as such we felt it more appropriate to include as supplemental information since we are essentially re-purposing an already-available data source.*

In addition, I found the discussion section not very well written and organized. I think the interpretation of the results could be clearer and more convincing. They, in my opinion, do not do a satisfactory job at answering basic questions that come to mind after reading the results section (see in comments below).

*Thank you, this sentiment was also brought up by reviewer #1 and as with our previous comments we have reorganized the manuscript to create a combined “Results and Discussion” section that pairs the data with their corresponding interpretations. This should be more fluid in presentation and correlate more directly the points raised.*

The abstract as well as the introduction describe the study sites and the context thoroughly, but the abstract does not emphasize the results and the main conclusions of their work, which in my opinion should be clarified. I also think that the results could have been discussed more thoroughly and in a clearer way. For instance, the fDOM results are barely discussed.

*We appreciate this compliment on the abstract and introduction! We have updated the abstract and clarified the main conclusions of the study, and changed the title to clarify the main findings. This should now be a more direct presentation of data. As mentioned earlier, we merged results and discussion sections, which should clarify the presentation.*

Here are my comments for each section of the manuscript.

Abstract:

The first paragraph could be shortened (in my opinion, only the first and last sentences should be conserved) and the authors should put more emphasis on their results and conclusions. Several sentences are very long and could be split to clarify the message.
The writing could be improved. Some statements are not entirely supported by the results or not explained well enough.

*Thank you for this comment, we have updated the abstract accordingly, removing text based on reviewer #2 suggestions and clarifying based on comments provided by reviewer #1. It is now a single paragraph. We emphasized the seasonal DOC characteristics, fluorescence data, and combined NEE/DOC flux, which we believe are supportive of the study’s hypotheses (that have been re-stated and clarified in the methods section in response to reviewer #1)*

L. 18-21: Both aqueous and gaseous C processes are key drivers of food webs and climate feedbacks in these ecosystems, regardless permafrost or glaciers are melting or not. This sentence is clumsy. The second part of the sentence (“as northern…”) is redundant with the first part. It is excessively long for the message it conveys.

*This sentence has been removed, thank you for the input.*

L. 30. DOC “inputs” or “outputs”?

*Updated to “exports”, this was noted by the other reviewer as well, thank you for catching it.*

L. 31-35. This sentence is too long. Split before “and fluorescence…” *Updated as suggested.*

L. 31. “depended on seasonal subsurface flowpath structure”. Not very clear. What do you mean exactly?

*We realize this statement could be interpreted as a confusing run-on; it was eliminated and is clarified in the following sentence.*

L. 35-38. “DOC fluxes…”. This sentence is too long and not clear. If there is a positive relationship between NEE and DOC outputs state it clearly. “revealing” is not appropriate. The first part of the sentence does not reveal anything like that. Of course the C that is fixed by the vegetation is internally processed. How couldn’t it be?

*We reworded “revealing” to “indicates” and eliminated words relating to internal processing.*

L. 40-42. Not supported by the data.

*Reworded to a general statement about the importance of DOC fluxes.*

Introduction

Overall, I don’t think the introduction is very well organized and written. Some sentences are too long (be careful to not overuse “as” - e.g., L.79-80) and ambiguous.
There should be a logical flow between the paragraphs, which is not always the case here. The authors should insist on the importance of the study on a climate change context.

*Thank you for this feedback, we have removed excessive use of the word “as”. We have re-visited the introduction with comments from both reviewers in regards to logical flow behind paragraphs. We agree completely that the importance of the study is in a climate change context and have highlighted that wherever possible.*

L. 61-62. No capital letters for boreal forest. I don’t really like the way you use “most important” to compare biomes. What about “the boreal forest plays a large role in global C cycle due to …”

*Updated as suggested, removed “important” and now the sentence reads, “The boreal forest is an important biome because of its large role in the terrestrial carbon cycle”*

L. 64. Planet’s

*Updated.*

L. 66. All ecosystems have gaseous and aqueous fluxes. Boreal forests are characterized by large DOC fluxes. Rewrite the sentence.

*The sentence has been rewritten to eliminate wording that makes this biome appear more important than others. “The boreal forest C cycle is comprised of both gaseous fixation and loses (i.e. CO2 and CH4) and dissolved fluxes…”*

L.71. “for” instead of “in”.

*Updated.*

L. 80. “increasingly”

*Updated.*

L.81. Why “decreased river flows”? Precipitation is not expected to decrease in the area and permafrost and glaciers thaw may increase river flow. I may have missed something. Clarify.

*Thank you for this question, we updated this sentence in the manuscript to clarify. The hydrograph is driven by glacial melt, and as glacial melt nears completion, discharge will decrease.*

L. 91-93. This sentence is not clear. I think you should describe the consequences of the previous sentences.

*Updated to indicate consequences of lower glacial melt-derived DOC and increased vegetation-derived DOC.*

L. 94-95. This sentence is ambiguous. Do you mean that terrestrial vegetation provides stream water with C directly (without passing through the soil)? What is the substrate you’re talking about? SOC or plant litter?

*Clarified these statements to note that vegetation contributes to DOC outputs directly and also has a role in SOC formation, which is relevant to DOC exports and throughflow/storage.*

L. 96. Litter inputs to what? Rivers or SOC?

*Updated to indicate litter inputs are relevant to both riverine DOC and SOC.*

L. 98. “to” DOC

*Fixed.*

L. 102. Why are gaseous C fluxes particularly more complex than in other ecosystems? Because of CH4 emissions due to anoxic conditions in the soil? explain.

*Removed wording that may give an impression of this system being more complex than others.*

L. 117. It partitions…

*This sentence was deleted in response to the previous comments from reviewer #1.*

L. 120. Briefly explain the nature and origin of fulvic acids; that FA differ depending on their origin and biogeochemical processes; that FA coming from plant litter and the soil have a higher degree of aromaticity as compared to microbially-derived FA; that we can estimate the relative contributions of FA sources by measuring FI. Some of this info is in your M&M section.

*In response to reviewer #1 comments on the broad presence of DOC fluorescence literature, we kept the introduction free of further background information on the topic. However, as this reviewer points out, we provide a description of the calculation of microbially-derived vs. terrestrial-derived FA in the methods section.*

L. 127 and L. 132. Dissolved “organic” C

*Updated.*

Materials and methods

Sampling was well performed. This part is clear and well described.

*Thank you for this compliment on our clear descriptions of sampling and other methods!*

A short explanation of the meaning of fDOM values or why they performed this type of analysis could be added.

*Updated to include short explanation on fDOM and why used.*

What kind of stats was performed on DOC seasonal trends? Mann-Kendall tests are commonly used.

*We elected to performed ANOVA tests on DOC seasonal trends to facilitate comparisons between sites. This does assume linearity (an assumption of our tests where p<0.05 indicate is valid). This is mentioned at the end of the methods section, and in order to not detract the reader from the comparisons we note that p values refer to this. When no linear trend was valid, we used seasonal mean values compared via student’s t-test.*

Results

Fig 2 and 4. These figures are not very clear. I would rather use lines than dots. It would help visualizing seasonal trends.

*Reformatted these figures as suggested to include lines. Agreed, this makes trends easier to visualize.*

Fig. 2B. Why isn’t delta 18O expressed in per mil?

*This was a typo, thank you for catching it. Updated to standard “delta” notation.*

Fig. 7. The x- and y-axes are not readable

*Fig 7 was replaced with a different fluorescence figure, which should have sufficient resolution to visualize.*

Why is there no title for the first paragraph?

*Results and Discussion are now merged and paragraphs have titles.*

Results from MCWT are not presented in the text.

*The new format of the combined results/discussion incorporates discussion into the results, which includes MCWT*

L. 277. The first two d18O values of May are high at YC. Mention it.

*Updated the manuscript to mention these high values.*

L. 283. Why cms and not m3 s-1?

*Respectfully, cms is “cubic meters per second” and consistently reported across the literature.*

L. 285. It would be insightful to display precipitation and temperature data to verify whether stream flow correlated with precipitation and/or snow melt resulting from high temperatures.

*Agreed; as with the previous comments, precip and temperature data are available in the supplemental materials section. We included the supplemental figure captions in the manuscript as requested by the editorial office.*

L. 298. Negatively correlated.

*Updated, thank you for catching this.*

L. 299. The trends are indeed similar, i.e. decrease from May to June, but the correlation is not obvious.

*Noted.*

L. 301. I agree that it correlates well in 2012 but not in 2013. It seems that there is a time lag.

*Noted.*

L. 301-302. The diurnally variation in fDOM is not visible from this figure.

*Thank you for mentioning this, we describe the diurnal variation of fDOM in the text body. Respectfully, this data is also made available with the manuscript should any reader wish to visualize the figure differently. We elected to prioritize the seasonal trends visually.*

L. 307-308. When does it correlated positively and negatively?

*This sentence refers to several statements that immediately follow; we have included a semicolon to link the two statements.*

L. 316-317. FI values are always >1.5. which -according to what you write in the M&M section- means that all DOC is from microbial origin.

*This section has been updated to expand the discussion on terrestrial and microbial -derived fluorescence character. We correlate PARAFAC components to HIX data and draw comparisons to other studies in the OpenFluor database.*

L. 328. Does “this” refer to “the combination of low FI and HIX”?

*Deleted the word “this” to clarify we are referring to seasonal fluorescence trends at this site.*

L. 337. “a” strong “sink”

*Updated.*

L. 338. “changed”

*Updated.*

L. 341. There are additional spaces after “at” and “s-1”.

*Fixed, thank you.*

Discussion

The section “seasonality of DOC export” is poorly written and not clear. The interpretation of the results is weak and no references are cited. The links between the different analyses (e.g. DOC, d18O, FI, HIX and conductance) should be discussed more deeply. One way to do that could be a reduction of the number of sections. The questions that come to mind after reading the results section are: Why is there a DOC concentration peak in May? Why is it higher at MAYC than at YNGC? Where does this DOC come from? Why is fDOM positively correlated with DOC flux? Why is fDOM positively correlated with Q in 2012 and negatively in 2013? Etc. The discussion should clearly answer these questions.

*Thank you for this feedback; together with the comments from reviewer #1, we combined the Discussion into a “Results and Discussion” with less sections. This provides direct linkages from study results to our interpretations, which may have not been clear before, or may have jumped around too much from topic to topic.*

L. 353. “North” America. The first sentence could be split after “North America”.

*Sentence updated.*

L. 357. What do you mean by “patterns”?

*We refer to the patterns in the following sentences; this word could be seen as superfluous and was deleted.*

L. 364. Riverine C what? Stocks? Fluxes?

*Updated to include “stocks and fluxes”, also that riverine C may “change” over time.*

L. 367. Is the amount of nutrients stored in the snow cover significant? What kind of nutrients are you talking about? Please, clarify this point.
L. 369. What about removing “however” and replace it by “which constitutes an impermeable …”. Do you mean that most DOC leached in May (during snow melt) comes entirely from the snow pack? Where does this DOC come from? Develop.

*(Similar comments combined) Thank you for pointing this out, we have updated and developed the statement to clarify we are referring to water-soluble labile carbon flushed from the upper-most soil layers. We assume the reviewer is referring to the word “moreover” instead of “however” since the latter is not present in the referenced line…we replaced “moreover” with “which constitutes and impermeable”… as we believe is being suggested.*

L. 371-373. This sentence is too long and contains too many “as”.

*Separated into two separate sentences and removed the word “as” in several places.*

L. 374. What are these nutrients with high specific conductance?

*Good question, we assumed SpCond is related to nutrient content but do not have data to support that. We clarified the statement to indicate, “ionized species associated with specific conductance”*

L. 375. The influence of precipitation events and subsurface mechanisms you’re talking about here is not clear.

*Clarified this sentence to read as follows, “With warming temperatures and a deepening active layer, we would expect an increased export resembling this chemical character”. The chemical character is referenced to SpCond and fluorescence readings in the preceding sentences.*

L. 386-390. The cause of the absence of trend in d18O in 2013 is not clear.

*We also do not understand why there was an absence of a trend; our best guess would be that an early snowmelt had a role.*

L. 393-394. Explain why. On average, the DOC export seems however as high in 2013 as in 2012.

*This is an astute observation that DOC export was similar for both years. We postulate on this in the paragraph, for 2013, “a combination of delayed, rapid snowmelt with an onset of low precipitation and warm air temperatures from May-July”*

L. 398. The high Q in May 2013 is due to a rapid snow melt. It was associated with low conductivity, low d18O and fDOM values, suggesting that DOC comes almost entirely from the snow pack. Can you explain why the snow pack contains so much DOC and where it comes from?

*We updated clarification in Results and Discussion section that this may not be due exclusively to the snowpack, but a combination of factors that include the flushing of landscape during a time of shallow active layer depth.*

L. 420. You could provide more information regarding biogenic and terrestrial DOC.

*The section on fluorescence has been extensively revised and updated to include information on biogenic and terrestrial DOC. We have included new discussion on our new PARAFAC model, including matches to known fluorescent components, matches to OpenFluor database and relations to other literature that identified similar DOC fluorescence.*

L. 424. Explain that the DOC output in May is more terrestrially-derived because microbial activity is low due to low temperatures. The DOC that’s released from the snow pack is not recycled by soil microorganisms.

*Thank you for this helpful suggestion! We have added it to the text.*

L. 437. Why does the throughfall C result in more humified DOC?

*We added text to clarify that Young Creek drains a higher elevation basin, much of which is above treeline.*

L. 441-443. I agree with this statement but it is not clear how your data support it. Develop.

*When combined in the Results/Discussion section along with new text on fluorescence character, this statement is now more fully-developed to indicate that aliphatic compounds of biogenic origin are liberated under a scenario of increased active layer depth.*

L. 444-455. This paragraph is very descriptive. It should be moved to the results section. As mentioned earlier, the resolution of the figure is too low for the x and y labels to be readable.

*Thank you for the compliment on this paragraph! It has been compiled into the fluorescence section of “Results and Discussion”*

L. 469. Your values are therefore very low as compared to those reported in the literature. How do you explain that?

*Updated text to indicate we believe low relief and dense standing biomass contribute to the low values compared with other studies.*

L. 481-484. C fixation would be indeed lower but as you mentioned before (L. 470), the DOC efflux would also be lower.

*Thank you for noting this. We included this information in the body of the text.*

L. 485-498. The last paragraph is good. In my opinion, the conclusion mentioned L.488, i.e. the watershed is C conservative, it is the main take home message and should appear in the title. The high seasonality of nutrients fluxes in boreal catchment is not new.

*Thank you for this suggestion; we have update the title accordingly. Thank you also for the compliment on the last paragraph!*

Conclusion

The conclusion is good but these points were in my opinion not developed enough in the discussion.

*Thank you for the compliment on the conclusion and we thank you for the feedback that allowed us to revise and develop the discussion more fully.*
